# Complement C3a activates osteoclasts by regulating the PI3K/PDK1/SGK3 pathway in patients with multiple myeloma

**DOI:** 10.20892/j.issn.2095-3941.2020.0430

**Published:** 2021-08-15

**Authors:** Fengjuan Jiang, Hui Liu, Fengping Peng, Zhaoyun Liu, Kai Ding, Jia Song, Lijuan Li, Jin Chen, Qing Shao, Siyang Yan, Kim De Veirman, Karin Vanderkerken, Rong Fu

**Affiliations:** 1 Department of Hematology, Tianjin Medical University General Hospital, Tianjin 300052, China; 2 Department of Hematology and Immunology-Myeloma Center Brussels, Vrije Universiteit Brussel, Brussels 1090, Belgium

**Keywords:** Multiple myeloma, complement C3a, osteoclasts, PI3K/PDK1/SGK3 pathways, SGK inhibitor

## Abstract

**Objective:**

Myeloma bone disease (MBD) is the most common complication of multiple myeloma (MM). Our previous study showed that the serum levels of C3/C4 in MM patients were significantly positively correlated with the severity of bone disease. However, the mechanism of C3a/C4a in osteoclasts MM patients remains unclear.

**Methods:**

The formation and function of osteoclasts were analyzed after adding C3a/C4a *in vitro*. RNA-seq analysis was used to screen the potential pathways affecting osteoclasts, and the results were verified by Western blot, qRT-PCR, and pathway inhibitors.

**Results:**

The osteoclast area per view induced by 1 μg/mL (mean ± SD: 50.828 ± 12.984%) and 10 μg/mL (53.663 ± 12.685%) of C3a was significantly increased compared to the control group (0 μg/mL) (34.635 ± 8.916%) (*P* < 0.001 and *P* < 0.001, respectively). The relative mRNA expressions of genes, OSCAR/TRAP/RANKL/cathepsin K, induced by 1 μg/mL (median: 5.041, 3.726, 1.638, and 4.752, respectively) and 10 μg/mL (median: 5.140, 3.702, 2.250, and 5.172, respectively) of C3a was significantly increased compared to the control group (median: 3.137, 2.004, 0.573, and 2.257, respectively) (1 μg/mL *P* = 0.001, *P* = 0.003, *P* < 0.001, and *P* = 0.008, respectively; 10 μg/mL: *P* < 0.001, *P* = 0.019, *P* < 0.001, and *P* = 0.002, respectively). The absorption areas of the osteoclast resorption pits per view induced by 1 μg/mL (mean ± SD: 51.464 ± 11.983%) and 10 μg/mL (50.219 ± 12.067%) of C3a was also significantly increased (33.845 ± 8.331%) (*P* < 0.001 and *P* < 0.001, respectively) compared to the control. There was no difference between the C4a and control groups. RNA-seq analysis showed that C3a promoted the proliferation of osteoclasts using the phosphoinositide 3-kinase (PI3K) signaling pathway. The relative expressions of PIK3CA/phosphoinositide dependent kinase-1 (PDK1)/serum and glucocorticoid inducible protein kinases (SGK3) genes and PI3K/PDK1/p-SGK3 protein in the C3a group were significantly higher than in the control group. The activation role of C3a in osteoclasts of MM patients was reduced by the SGK inhibitor (EMD638683).

**Conclusions:**

C3a activated osteoclasts by regulating the PI3K/PDK1/SGK3 pathways in MM patients, which was reduced using a SGK inhibitor. Overall, our results identified potential therapeutic targets and strategies for MBD patients.

## Introduction

Multiple myeloma (MM) is a hematological malignancy resulting from uncontrolled proliferation of plasma cells in the bone marrow. Myeloma bone disease (MBD) is the most common complication of MM, with approximately 80%–90% of patients showing bone lesions at diagnosis^1^. MBD mainly manifests as hypercalcemia, severe osteoporosis, severe bone pain, osteolytic lesions, pathological fractures, and vertebral compression fractures, which have negative effects on both the quality of life and survival of patients^2^. The pathogenesis of MBD is mainly a result of the formation of osteoclasts activated by MM cells or stromal cells, which leads to the enhancement of bone resorption function, accompanied by the inhibition of osteoblast function, which eventually leads to an imbalanced bone metabolism^1,3^. Extensive research on the function of osteoclasts has shown that the mechanism of osteoclast activation is complex, and plays a critical role in the pathogenesis of MBD^4^.

The complement system has an important relationship with bone development and homeostasis^5^. Complement proteins, including C3, factor B, C5, and C9, are found in the growth plate during bone development^6^. C1s are expressed by hypertrophic chondrocytes in the ossification center of the femur, but not by normal articular cartilage cells^7,8^. Ignatius et al.^9^ found that the C3 and C5 complement components, the C3aR and C5aR anaphylatoxin receptors, the MCP (CD46), DAF (CD55) complement regulators, and MACIF (CD59) were expressed in differentiated and undifferentiated mesenchymal stem cells (MSCs) and osteoblasts. Moreover, osteoclasts express MACIF (CD59), C3aR, C5aR, and C3^9^, and the formation of osteoclasts is regulated by C3 (C3a), C5a, C3aR, and C5aR^10–12^. In addition, Teo et al.^13^ found that C1q was produced by osteoclasts, and was responsible for enhancing osteoclast development.

In the present study, we analyzed correlations of serum levels of C3/C4/C4a in both newly diagnosed multiple myeloma (NDMM) patients and healthy controls, and bone disease stages, with the number of osteoblast precursors, osteoclast precursors (OCPs), and biological indicators of MBD. In a previous study, we reported the effects of C3a/C4a on the occurrence of MBD^14^, and found that serum levels of C3, C4, and C4a in NDMM patients were significantly positively correlated with the severity of bone disease, the number of OCPs, and the levels of the C-terminal cross-linking telopeptide biomarker of type I collagen/tartrate resistant acid phosphatase isoform-5b, which is related to bone destruction. We therefore proposed that serum levels of C3 and C4 may be sensitive biomarkers of myeloma bone disease, so we characterized the effects and potential mechanisms of C3a/C4a on osteoclasts in patients with MM.

## Materials and methods

### Patients and samples

The study cohort included 124 NDMM patients. All patients were in-patients at the Hematology Department of Tianjin Medical University General Hospital, from May 2017 to May 2019. Patients were diagnosed according to International Myeloma Working Group updated criteria for MM^15,16^. The clinical characteristics of the patients are shown in **Table 1**. This study was approved by the Ethical Committee of Tianjin Medical University (Approval No. IRB2020-WZ-018). Samples (10 mL) of bone marrow were obtained from all NDMM patients after obtaining written informed consent from the participants.

**Table 1:** Baseline characteristics of patients

Characteristics	Newly diagnosed multiple myeloma patients (*n* = 124) (%)
Gender	
Male	78 (62.903)
Female	46 (37.10)
Age (median)	64
Range	34˜87
R-ISS stage	
I	15 (12.10)
II	26 (20.97)
III	83 (66.93)
M-component type	
IgG	62 (50.00)
IgA	25 (20.16)
IgM	1 (0.8)
Light chain only	26 (20.97)
Nonsecretory	10 (8.07)
_2_-microglobulin	
< 5.5 mg/L	41 (33.06)
≥ 5.5 mg/L	83 (66.94)
Ca	
> 2.75 mmol/L	30 (24.19)
Serum creatinine	
< 177 μmol/L	80 (64.52)
≥ 177 μmol/L	44 (35.48)
Hb	
< 100 g/L	85 (68.55)
≥ 100 g/L	39 (31.45)

R-ISS: Revised International Staging System; Hb: hemoglobin.

### Osteoclast cultures

Bone marrow mononuclear cells (BMMCs) were isolated from the bone marrow of patients using Ficoll-Paque Plus solution (Amersham Biosciences, Chalfont St. Giles, UK). Isolated BMMCs were incubated in α-MEM medium supplemented with 10% fetal bovine serum (FBS), 100 U/mL penicillin, and 100 mg/mL streptomycin in the presence of recombinant human receptor activator for nuclear factor-κ B ligand (RANKL) (150 ng/mL; Miltenyi Biotec, Sunnyvale, CA, USA) and recombinant human macrophage colony-stimulating factor (M-CSF) (50 ng/mL; Miltenyi Biotec, USA), followed by seeding in a 24-well plate at 1 × 10^6^ cells/well, as described previously^17^. The cells of each patient were divided into 3 wells with different concentrations of recombinant human complement C3a (Emd Millipore, Burlington, MA, USA) at 10 μg/mL, 1 μg/mL, or 0 μg/mL DMSO), according to studies on the effect of human C3a on osteoblasts, osteoclasts, and MSCs^9,18^. The complement C4a experiment was similar, with a concentration of C4a (Fitzgerald, Crossville, TN, USA) of 10 μg/mL, 1 μg/mL, or 0 μg/mL; the concentration was set according to the serum level of C4a measured by an ELISA in NDMM patients. To study the effect of the SGK inhibitor (EMD638683) on osteoclasts, the concentration of EMD638683 (MedChemExpress, Monmouth Junction, NJ, USA) was set to 10 μM, 1 μM, or 0 μM, according to previous studies on EMD638683^19,20^. The 24-well plate was cultured for 14 days at 37 °C in a humidified incubator containing 5% CO_2_. Culture media with cytokines, recombinant human complement proteins, or EMD638683 were replaced every 3 days.

### TRAP staining

Osteoclasts were identified using a TRAP staining kit (Sigma-Aldrich, St. Louis, MO, USA), according to manufacturer’s instructions. Briefly, 0.5 mL of Fast Garnet GBC base solution and 0.5 mL of sodium nitrite solution were added to a tube and mixed by gentle inversion for 30 s. Then, 100 μL of the resulting mixture was mixed with 4.5 mL of deionized water, 50 μL of naphthol AS-BI phosphate solution, 200 μL of acetate solution, and 100 μL of tartrate solution to a final volume of 10 mL in a tube. The 24-well plates with osteoclasts were fixed by immersing in fixative solution for 30 s. Finally, 500 μL of the mixed solution was added to each well and incubated 10 min in a water bath at 37 °C in the dark. After 10 min, the plates were rinsed thoroughly in deionized water and evaluated microscopically. Purple multinucleated cells (≥ 3 nuclei) were defined as osteoclasts.

### Quantitative real-time PCR

Quantitative real-time PCR was performed as described previously^21^. RNA was extracted from osteoclasts using TRIzol reagent (Invitrogen, Carlsbad, CA, USA) and mRNA expression was quantified using the Bio-Rad iQ5 Real-time system (Bio-Rad, Hercules, CA, USA). The primer sequences of osteoclast-associated genes RANKL/OSCAR/TRAP/Cathepsin K and related genes of the C3a activating the osteoclasts pathway are shown in **Supplementary Table S1** and include: SGK3/PIK3CA/POSTN/COL1A1/COL1A2/CREB1/MDM2/IKBKG/NTRK2/CDK2/TCL1A/PDK1/GAPDH.

SYBR Green (Invitrogen) was used as a double-strand DNA-specific dye. The amplification of RANKL/OSCAR/TRAP/Cathepsin K/SGK3/PIK3CA/POSTN/COL1A1/COL1A2/CREB1/MDM2/IKBKG/NTRK2/CDK2/TCL1A/PDK1 was conducted over 45 cycles at 95 °C for 30 s and 95 °C for 5 s, followed by extension at the temperatures shown in **Supplementary Table S2**. Glyceraldehyde 3-phosphate dehydrogenase (GAPDH) was used as the housekeeping gene to standardize the targeted mRNA expression. The levels of RANKL/OSCAR/TRAP/Cathepsin K/SGK3/PIK3CA/POSTN/COL1A1/COL1A2/CREB1/MDM2/IKBKG/NTRK2/CDK2/TCL1A/PDK1 were calculated using the 2^-ΔΔCt^ method [(Ct, target gene Ct, GAPDH) sample - (Ct, target gene Ct, GAPDH) control] after normalizing the data to GAPDH mRNA expression.

### Resorption pit assay

Isolated BMMCs were incubated in α-MEM medium supplemented with 10% FBS, 100 U/mL penicillin, and 100 mg/mL streptomycin in the presence of recombinant human RANKL (150 ng/mL), recombinant human M-CSF (50 ng/mL), recombinant human complement C3a/C4a, or EMD638683, followed by seeding on bovine cortical bone slices in a 24-well plate at 1 × 10^6^ cells/well for 14 days at 37 °C in a humidified incubator containing 5% CO_2_. Culture media with cytokines, recombinant human complement proteins, or EMD638683 were replaced every 3 days. After culturing, the bovine cortical bone slices were fixed with 2.5% glutaraldehyde for 7 min, washed 3 times by sonication in 0.25 M aqua ammonia for 5 min, and subjected to ethanol gradient dehydration, drying, and spraying before observation using scanning electron microscopy (SEM).

### RNA-seq analysis

Total RNA was extracted from the cell samples for use in RNA-seq analysis. The RNA samples were converted into individual cDNA libraries and generated using the BGISEQ-500 platform of the Beijing Genomics Institute (BGI) (Beijing, China)^22,23^. All of the generated raw sequencing reads were filtered to obtain clean reads before storing in the FASTQ format^24^. Clean reads were aligned to reference genes and genomes using Bowtie2 and HISAT, respectively^25,26^. The expression levels of the genes were normalized to FPKM using RSEM^27^. Genes were selected using the criteria of differentially expressed genes (DEGs) between the C3a group and control with a fold-change ≥ 1.5 and adjusted *P-*value ≤ 0.05. The relative enrichment and pathway annotation of genes for various functional associations were determined using the Kyoto Encyclopedia of Genes and Genomes (KEGG) pathway database.

### Western blot

Western blot analysis was performed as described previously^28^ to evaluate the PI3K/PDK1/SGK3/p-SGK3/AKT/p-AKT protein concentrations in the osteoclasts supplemented with recombinant human complement C3a or EMD638683. The cells were lysed on ice in a lysis buffer containing a phenylmethylsulfonyl fluoride protease inhibitor (Sigma-Aldrich), phosphatase inhibitor (Cell Signaling Technology, Danvers, MA, USA), and RIPA lysis buffer (Sigma-Aldrich) configured in a proportion of 1:1:100. The protein concentration was determined using a bicinchoninic acid (BCA) protein assay kit (Thermo Fisher Scientific, Waltham, MA, USA). The extracted proteins (30 μg/lane) were separated using SDS/PAGE (12% gels). The proteins were subsequently transferred to nitrocellulose membranes (Bio-Rad Laboratories, Hercules, CA, USA), which were blocked using 5% skim milk by incubating for 1 h at room temperature. The membranes were then incubated with primary antibodies against PI3K/PDK1/SGK3/p-SGK3/AKT/p-AKT/GAPDH [1:1000 dilution in 5% bovine serum albumin (BSA); Cell Signaling Technology] overnight at 4 °C. The membranes were washed with Tris-buffered saline containing 0.1% Tween-20 and incubated with horseradish peroxidase-conjugated anti-rabbit IgG sheep antibody (1:5000 dilution in 5% BSA; Abcam, Cambridge, MA, USA) for 1 h at room temperature. The protein bands were visualized using the hypersensitive ECL chemiluminescence kit (Cell Signaling Technology).

### Statistical analysis

Data are expressed as numerical variables. The osteoclast area, absorption area of osteoclast resorption pit per view, and the relative gray scale of related protein expressions are expressed as the mean ± standard deviation (SD). The unpaired *t*-test and one-way analysis of variance were used to analyze the significance between different groups. The expressions of target genes are expressed as the median. Comparison between data were conducted using a nonparametric test. SPSS statistical software for Windows, version 21.0 (SPSS, Chicago, IL, USA) was used to conduct the statistical analysis. *P* < 0.05 was considered statistically significant.

## Results

### Complement C3a significantly promoted the formation and function of osteoclasts, while complement C4a did not

To evaluate the effect of C3a/C4a on osteoclasts in NDMM patients, we observed the formation and function of osteoclasts in different concentrations of C3a and C4a (1 μg/mL and 10 μg/mL).

**Figure 1:**
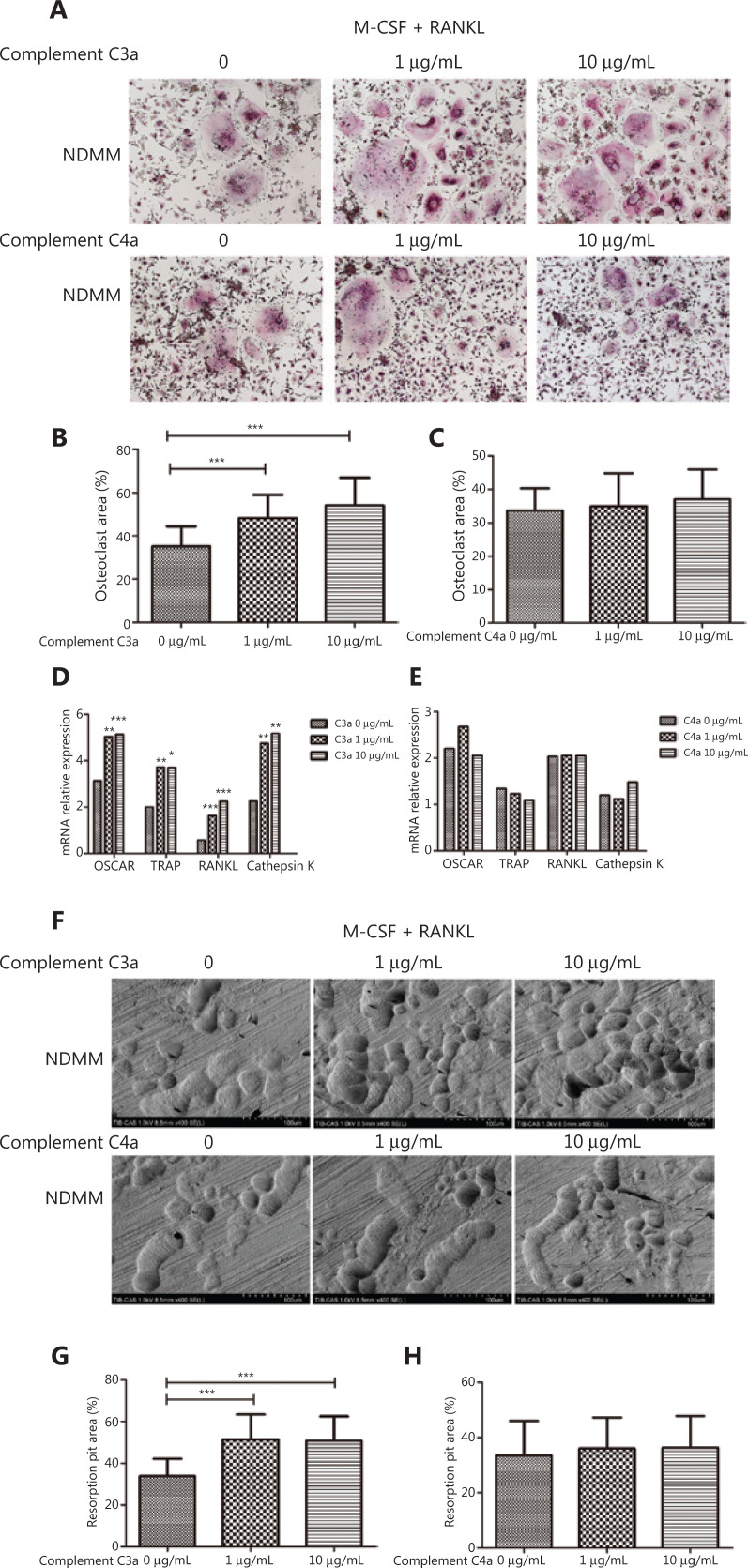
Complement C3a significantly promoted the formation and function of osteoclasts, while complement C4a did not. (A) The osteoclasts areas observed by TRAP staining per view induced with 1 μg/mL and 10 μg/mL of C3a/C4a. Original magnification: 100× (bar: 100 μm).(B) The osteoclasts areas per view induced by 1 μg/mL (mean ± SD: 50.828 ± 12.984%) and 10 μg/mL (53.663 ± 12.685%) of C3a were significantly increased when compared to the control group (0 μg/mL) (34.635 ± 8.916%) (*P* < 0.001 and *P* < 0.001, respectively) (*n* = 30). (C) There was no difference among the osteoclasts areas between the C4a and the control group (*n* = 15). (D) The relative expressions of mRNAs of genes *OSCAR/TRAP/RANKL*/cathepsin K induced by 1 μg/mL (median: 5.041, 3.726, 1.638, and 4.752, respectively) and 10 μg/mL (median: 5.140, 3.702, 2.250, and 5.172, respectively) of C3a was significantly increased compared to the control group (median: 3.137, 2.004, 0.573, and 2.257, respectively) (1 μg/mL: *P* = 0.001, *P* = 0.003, *P* < 0.001 and *P* = 0.008, respectively; 10 μg/mL: *P* < 0.001, *P* = 0.019, *P* < 0.001, and *P* = 0.002, respectively) (*n* = 30). (E) There was no difference between the relative expressions of genes *OSCAR/TRAP/RANKL*/cathepsin K between the C4a and the control group (*n* = 21). (F) The absorption areas of osteoclast resorption pit per views induced by C3a/C4a. (G) The absorption areas of osteoclast resorption pit per views induced by 1 μg/mL (mean ± SD: 51.464 ± 11.983%) and 10 μg/mL (50.219 ± 12.067%) of C3a was also significantly increased (33.845 ± 8.331%) (*P* < 0.001 and *P* < 0.001, respectively) compared to the control (*n* = 30). (H) There was no difference among the absorption areas of osteoclast resorption pits between the C4a and the control group (*n* = 15) (^*^P < 0.05, ^**^P < 0.01, and ***P < 0.001, respectively).

*In vitro*, the osteoclasts area per view from 30 patients induced by 1 μg/mL (mean ± SD: 50.828 ± 12.984%) and 10 μg/mL (53.663 ± 12.685%) of C3a was significantly increased compared to the control group (0 μg/mL) (34.635 ± 8.916%) (*P* < 0.001; *P* < 0.001) (**Figure 1 A and 1B**). There was no difference between 1 μg/mL (mean ± SD: 34.942 ± 9.920%) and 10 μg/mL (37.034 ± 8.964%) of the C4a and control groups (33.635 ± 6.639%) in 15 patients (**Figure 1 A and 1C**).

The relative mRNA expressions of the OSCAR/TRAP/RANKL/Cathepsin K genes from 30 patients were measured. The expressions of these genes induced by 1 μg/mL (median: 5.041, 3.726, 1.638, and 4.752, respectively) and 10 μg/mL (median: 5.140, 3.702, 2.250, and 5.172, respectively) in the C3a group was significantly increased compared to the control group (median: 3.137, 2.004, 0.573, and 2.257, respectively) (1 μg/mL: *P* = 0.001,* P* = 0.003, *P* < 0.001, and *P* = 0.008, respectively; 10 μg/mL: *P* < 0.001, *P* = 0.019, *P* < 0.001, and *P* = 0.002, respectively) (**Figure 1D**). There was no difference among the relative expressions of osteoclast-related genes (OSCAR/TRAP/RANKL/Cathepsin K, respectively) between 1 μg/mL (median: 2.672, 1.231, 2.056, and 1.115) and 10 μg/mL (median: 2.056, 1.084, 2.049, and 1.483) of the C4a *vs.* the control groups (median: 2.206, 1.341, 2.036, and 1.202) in 21 patients (**Figure 1E**).

The absorption area of the osteoclast resorption pit per view induced by 1 μg/mL (mean ± SD: 51.464 ± 11.983%) and 10 μg/mL (50.219 ± 12.067%) of C3a was also significantly increased (33.845 ± 8.331%) (*P* < 0.001 and *P* < 0.001) in 30 NDMM patients compared to the control group (**Figure 1F and 1G**). There was no difference among the absorption areas of the osteoclast resorption pits between 1 μg/mL (mean ± SD: 35.950 ± 11.284%) and 10 μg/mL (36.279 ± 11.427%) of the C4a *vs*. the control group (33.594 ± 12.452%) in 15 patients (**Figure 1F and 1H**).

Together, these results suggested that C3a at concentrations of 1 μg/mL and 10 μg/mL significantly promoted the formation, differentiation, and functioning of osteoclasts in MM patients, while C4a did not.

### Complement C3a promoted the formation and function of osteoclasts in NDMM patients by regulating the PI3K/PDK1/SGK3 pathway

**Figure 2:**
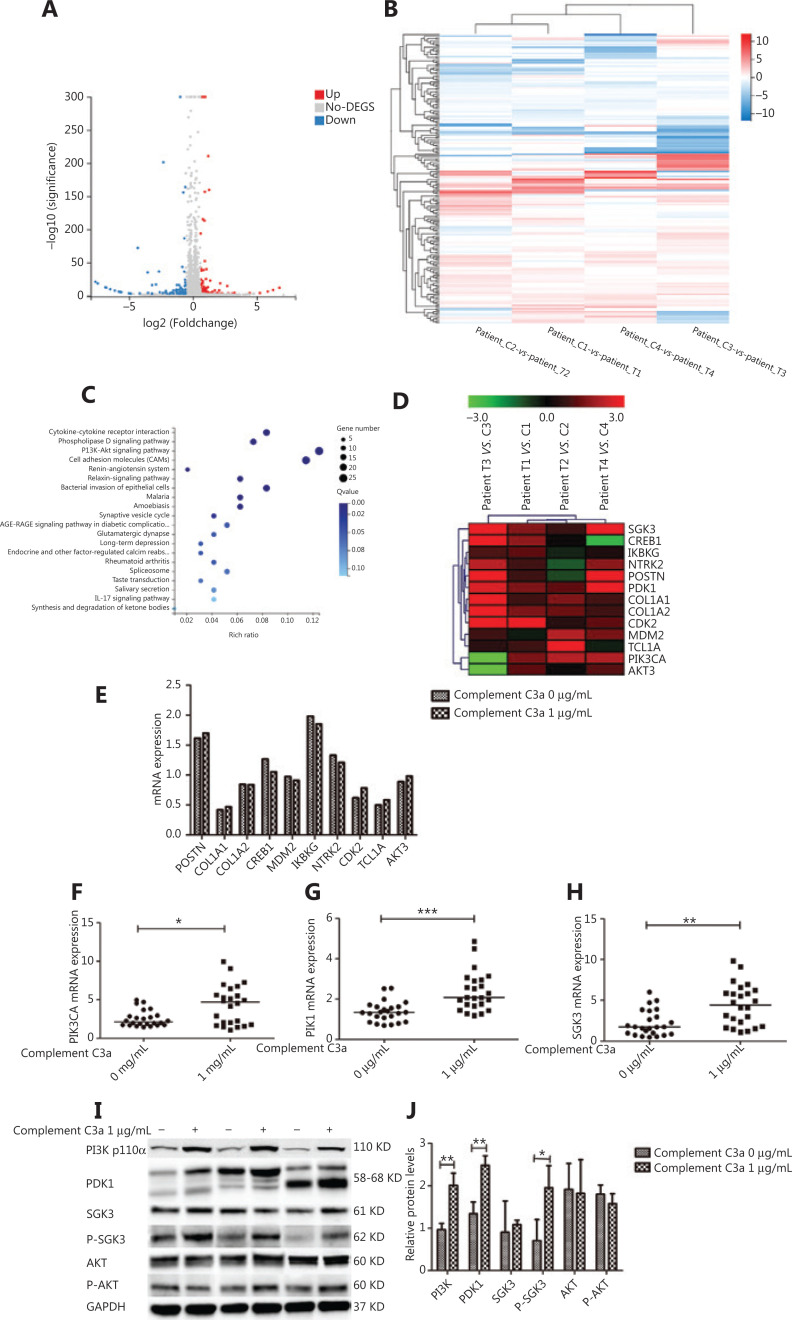
Complement C3a promoted the formation and function of osteoclasts in patients with newly diagnosed multiple myeloma (NDMM) by regulating the PI3K/PDK1/SGK3 pathway. (A) RNA-seq analysis was performed on the total RNA of osteoclasts in 4 patients with newly diagnosed MM treated with 1 μg/mL C3a and dimethylsulfoxide. There were 184 differentially expressed genes (≥ 1.5-fold change) between the C3a and the control group; 97 upregulated genes and 87 downregulated genes were shown in the volcanic map. (B) Differentially expressed genes (≥ 1.5-fold change) between the C3a and the control group are shown as a hot map. (C) The Kyoto Encyclopedia of Genes and Genomes pathway of differentially expressed genes (≥ 1.5-fold change) between the C3a and the control group was analyzed. (D) Selection of upregulated DEGs in the PI3K signaling pathways in at least 3 sequenced patients is shown as a heat map. (E) Upregulated differentially expressed genes were validated by qRT-PCR between the C3a (1 μg/mL) and the control group in 24 patients with NDMM. There was no statistically significant difference in the relative expressions of *POSTN/COL1A1/COL1A2/CREB1/MDM2/IKBKG/NTRK2/CDK2/TCL1A/AKT3* between the C3a and the control group. (F, G, H) The relative expression level of PIK3CA/phosphoinositide dependent kinase-1 (*PDK1*)/serum and glucocorticoid inducible protein kinase (*SGK3*) genes (median: 4.717, 2.078, and 4.428, respectively) in the C3a group (1 μg/mL) were significantly higher than in the control group (median: 2.135, 1.336, and 1.714, respectively) (*P* = 0.022, *P* < 0.001, and *P* = 0.001, respectively). (I) The expressions of PI3K p110α/Akt/p-Akt/PDK1/SGK3/p-SGK3 proteins in the PI3K signaling pathway were detected by Western blot. (J) The relative grayscale levels of PI3K p110α/PDK1/p-SGK3 protein (mean ± SD: 2.012 ± 0.286, 2.481 ± 0.228, and 1.956 ± 0.519, respectively) in the C3a group (1 μg/mL) were significantly greater than the control group (mean ± SD: 0.967 ± 0.147, 1.341 ± 0.278, and 0.709 ± 0.494, respectively) (*P* = 0.005, *P* = 0.005, and *P* = 0.039, respectively) (^*^P < 0.05, ^**^P < 0.01, and ^***^P < 0.001, respectively).

To further identify related pathways, RNA-seq analysis was performed on the patient-derived osteoclasts of 4 NDMM patients treated with 1 μg/mL C3a and dimethyl sulfoxide. There were a total of 184 DEGs (fold-change ≥ 1.5) between the C3a and control groups, including 97 upregulated genes, and 87 downregulated genes (**Figure 2A and 2B**). Based on KEGG pathway analyses, the top 4 groups were genes involved in cytokine-cytokine receptor interaction (the main genes included *CNTF/ CSF2/CSF3/IL-17A/CXCL10/CCL1/TNFSF12/TNFSF13/IFNA/IL-9*), the phospholipase D signaling pathway (the main genes included *PLA2G4B/IGFBP2/SHC1/SHC4/MS4A6A/GOLGA6L10/GOLGA8K/GPCR/RTK/IL-8*), the phosphoinositide 3-kinase (PI3K) signaling pathway (including the PI3K-Akt signaling pathways and AKT-independent PI3K signaling pathways) (the main genes included *PIK3CA/PDK1/SGK3/POSTN/COL1A1/COL1A2/CREB1/MDM2/IKBKG/NTRK2/CDK2/TCL1A/AKT3*), and cell adhesion molecules (CAMs) (the main genes included *HAPLN1/F13A1/ANGPTL2/CEACAM6/PTPRS/CLEC19A/ITGB1/ITGB2/SELE/NRCAM/CFPG2/SDC/PTPRF*) (**Figure 2C**). The PI3K signaling pathway mainly controls cell growth, transcription and translation, cell proliferation, cell movement, and glycogen metabolism, and is most likely to be related to osteoclast formation, differentiation, and maturation. DEGs on the PI3K signaling pathway that were upregulated in at least 3 patients were therefore selected and analyzed, as shown in the heat map (**Figure 2D**).

The upregulated DEGs were validated by qRT-PCR between the C3a and control group in 24 NDMM patients. There was no statistically significant difference with regards to the relative expressions of POSTN/COL1A1/COL1A2/CREB1/MDM2/IKBKG/NTRK2/CDK2/TCL1A/AKT3 between the C3a and control groups (**Figure 2E**). Fortunately, the relative expression levels of the *PIK3CA/PDK1/SGK3* genes (median: 4.717, 2.078, and 4.428, respectively) in the C3a group (1 μg/mL) were significantly higher than in the control group (median: 2.135, 1.336, and 1.714, respectively) (*P* = 0.022, *P* < 0.001, and *P* = 0.001, respectively) (**Figure 2F–2H**). We speculated that the promotional effect of C3a on osteoclasts in NDMM patients may have been related to upregulation of the *PIK3CAA*,* PDK1*, and *SGK3* genes.

The expression levels of PI3K p110α/Akt/p-Akt/PDK1/SGK3/p-SGK3 proteins in the PI3K signaling pathway were detected by Western blot. There was no significant difference in the relative grayscale of proteins Akt/p-Akt/SGK3 between the C3a (1 μg/mL) and control groups (**Figure 2I and 2J**). The relative grayscale levels of PI3K p110α/PDK1/p-SGK3 proteins (mean ± SD: 2.012 ± 0.286, 2.481 ± 0.228, and 1.956 ± 0.519, respectively) in the C3a group (1 μg/mL) were significantly higher than in the control group (mean ± SD: 0.967 ± 0.147, 1.341 ± 0.278, and 0.709 ± 0.494, respectively) (*P* = 0.005, *P* = 0.005, and *P* = 0.039, respectively) (**Figure 2I and 2J**).

The expression levels of PI3K and PDK1 (both upstream of SGK3 and Akt), and p-SGK3 proteins increased, while the expressions of Akt and p-Akt proteins did not change, suggesting that C3a promoted the formation, differentiation, and functioning of osteoclasts in MM patients by regulating the PI3K/PDK1/SGK3 pathway.

### Treatment with SGK inhibitor significantly inhibited the promoting effect of C3a on osteoclasts in NDMM patients

Ackermann et al.^19^ and Basnet et al.^20^ showed that the SGK inhibitor, EMD638683, could inhibit SGK3 up to 75% at a concentration of 1 μM. EMD638683 was therefore selected as the SGK3 inhibitor in this study.

**Figure 3:**
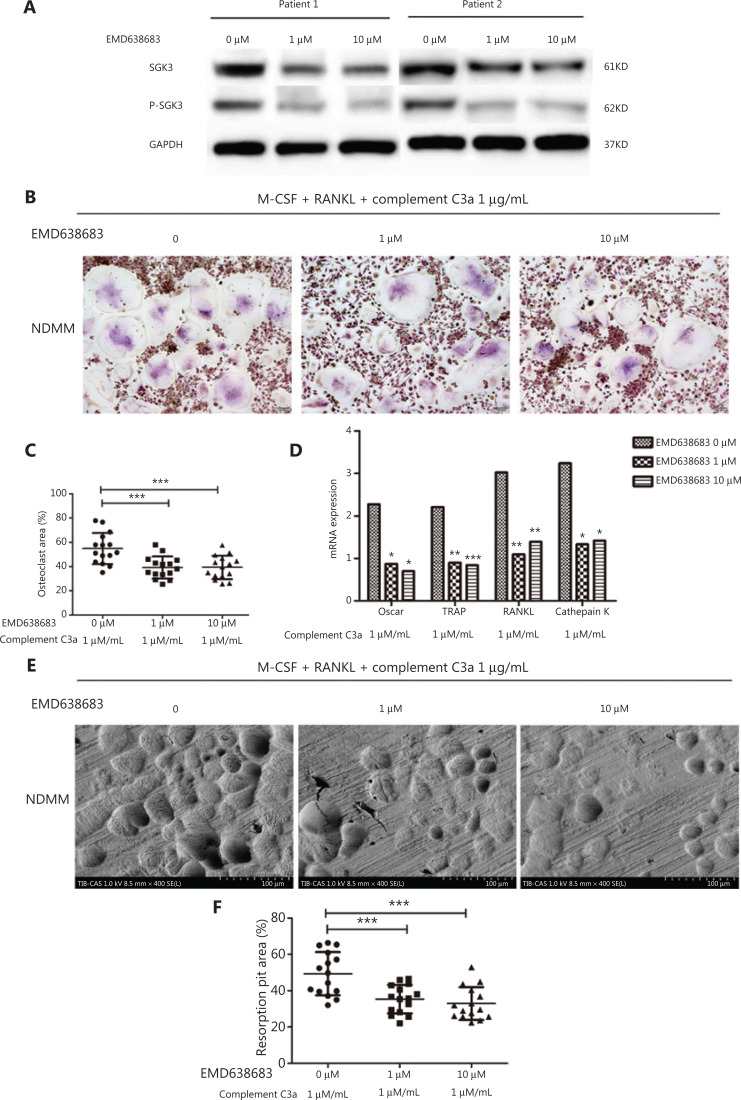
After treatment with a SGK inhibitor, the positive effect of C3a on osteoclasts in patients with newly diagnosed multiple myeloma was significantly inhibited. (A) The expressions of SGK3 and p-SGK3 proteins in the EMD638683 group were significantly lower than the control. (B, C) EMD638683 treatment significantly activated osteoclasts and reduced the osteoclasts area per view in the 1 μM (mean ± SD: 39.244 ± 9.089%) and 10 μM (39.299 ± 9.587%) EMD638683-treated groups when compared to the control group (0 μM) (54.884 ± 12.837%) (*P* < 0.001 and *P* < 0.001, respectively). (D) The relative expressions of osteoclast related genes *OSCAR/RANKL/TRAP*/cathepsin K in the 1 μM (median: 0.869, 1.097, 0.902, and 1.328, respectively) and the 10 μM (median: 0.703, 1.391, 0.843, and 1.418, respectively) EMD638683 groups were significantly decreased when compared to the control group (median: 2.270, 3.024, 2.208, and 3.237, respectively) (1 μM: *P* = 0.015, *P* = 0.002, *P* = 0.003, and *P* =0.015, respectively; 10 μM: *P* = 0.012, *P* = 0.006, *P* < 0.001, and *P* = 0.017, respectively). (E, F) The absorption areas per view of osteoclast resorption pits in the 1 μM (mean ± SD: 35.383 ± 7.794%) group and 10 μM of EMD638683 group (32.886 ± 8.993%) were significantly reduced compared to the control group (49.358 ± 11.856%) (*P* < 0.001 and *P* < 0.001, respectively) (^*^P < 0.05, ^**^P < 0.01, and ^***^P < 0.001, respectively).

The effects of EMD638683 on the expressions of SGK3 and p-SGK3 proteins in osteoclasts of NDMM patients were validated by Western blot. The results showed that the expressions of SGK3 and p-SGK3 proteins in the EMD638683 group were significantly lower than in the control group, confirming that EMD638683 inhibited SGK3 and p-SGK3 in the osteoclasts of NDMM patients (**Figure 3A**).

The SGK inhibitor (EMD638683) was then added to a culture system of osteoclasts activated by C3a (1 μg/mL) to evaluate whether the formation and function of osteoclasts were inhibited. Treatment with EMD638683 significantly reduced the osteoclasts areas per view in the 1 μM (mean ± SD: 39.244 ± 9.089%) and 10 μM (39.299 ± 9.587%) EMD638683-treated group, when compared to the control group (0 μM) (54.884 ± 12.837%) (*P* < 0.001 and *P* < 0.001, respectively) (**Figure 3B and 3C**). The relative expressions of the osteoclast-related genes *OSCAR/RANKL/TRAP*/cathepsin K in the 1 μM (median: 0.869, 1.097, 0.902, and 1.328, respectively) and 10 μM (median: 0.703, 1.391, 0.843, and 1.418, respectively) EMD638683 treatment groups were significantly decreased compared to the control (median: 2.270, 3.024, 2.208, and 3.237, respectively) (1 μM: *P* = 0.015, *P* = 0.002, *P* = 0.003, and *P* = 0.015, respectively; 10 μM: *P* = 0.012, *P* = 0.006, *P* < 0.001, and *P* = 0.017, respectively) (**Figure 3D**). The absorption areas per view of osteoclast resorption pits in the 1 μM (mean ± SD: 35.383 ± 7.794%) and 10 μM (32.886 ± 8.993%) EMD638683-treated groups were significantly reduced compared to the control group (49.358 ± 11.856%) (*P* < 0.001 and *P* < 0.001, respectively) (**Figure 3E and 3F**). Under the promotion of C3a, no difference was observed between the effects of 1 μM and 10 μM EMD638683 on the osteoclasts areas, relative expression of osteoclast-related genes, or absorption area of osteoclast resorption pits.

## Discussion

MM is a hematological malignancy caused by the proliferation of plasma cells resulting in multiple organ function impairment. Osteoclast activation and osteoblast inhibition results in metabolism imbalance in bones. Over 80% of MM patients suffer from bone disease to different degrees at diagnosis, which seriously affects the quality of life and their prognoses^29^. The study of the pathogenesis of MBD is key for the identification of sensitive biomarkers for early diagnosis and the identification of new therapeutic targets for MBD. The complement system is an important part of the immune system and is associated with bone reconstruction and destruction^5,30^. MBD is associated with an imbalance between bone remodeling and destruction. Whether the complement system is related to this imbalance has not yet been reported. We found that serum levels of C3, C4, and C4a in NDMM patients were significantly correlated with the severity of bone disease, as described previously^14^. Many studies have focused on the function of osteoclasts; however, the mechanism of osteoclast activation is complex, and plays a critical role in the pathogenesis of MBD^3,4^. In this study, we characterized the effects and potential mechanisms of C3a/C4a on osteoclasts in MM patients.

We showed that C3a significantly promoted the formation and function of osteoclasts in MM patients, while the C4a complement did not. The C3a complement is a common focal point of three complement activation pathways, and has been extensively studied in bone metabolism. Several studies have indicated that C3a activates osteoclasts, consistent with our results on the effects of C3a on osteoclasts in MM patients. Sato et al.^11^ found that the differentiation of osteoclasts in mouse bone marrow was inhibited after the addition of anti-C3 antibody. Using bone marrow cells from wild-type and C3-deficient mice (C3^−/−^), C3^−/−^ bone marrow cultures were found to generate fewer osteoclasts than wild-type marrow cells, with several features of osteoclast formation impaired in the bone marrow cultures of C3^−/−^ mice^10^. MacKay et al.^31^ showed that C3 deficiency reduced bone loss at ovariectomy and may improve mechanical properties. Moreover, Matsuoka et al.^32^ found that osteoclasts secreted C3a, which stimulated osteoblastogenesis and was involved in bidirectional communication between osteoblasts and osteoclasts.

No difference was observed between the 1 μg/mL and 10 μg/mL C3a groups regarding the effects on osteoclasts of MM patients. We hypothesized that C3a may function through the C3a receptor, which was already saturated at 1 μg/mL C3a. Schraufstatter et al.^18^ found that chemoattractants of C3a for human MSCs showed increased concentrations of 0, 30 nM, and 100 nM, and peaked at concentrations of 100 nM, before gradually decreasing at concentrations of 300 nM and 1 μM. This suggested that the concentration dependence on C3a for the activation of osteoclasts in MM patients may occur between 0 and 1 μg/mL.

The effects of C4a on bone metabolism have not been previously reported. However, Zheng et al.^33^ found that serum levels of C4 in patients with MM increased, suggesting that C4 was associated with the pathogenesis of MM. Dowling et al.^34^ used a label-free mass spectrometry-based methodology to evaluate serum samples of monoclonal gammopathy of undetermined significance, smoldering multiple myeloma, MM patients with no bone disease, and MM patients with high bone disease. They found that the level of C4a increased in high bone disease MM patients, and identified C4a as a novel candidate biomarker associated with bone disease. However, these results were only obtained from correlation studies and not from functional experiments *in vitro* and *in vivo*. Our study is the first to report the effects of C4a on osteoclasts in MM patients. The results suggested that C4a at concentrations of 1 μg/mL and 10 μg/mL could not promote the formation, differentiation, or function of osteoclasts in MM patients. This may be due to C4a affecting osteoclasts by acting on other complement proteins, or may be simply due to C4a inhibiting osteoblasts, with little effect on osteoclasts. Barnum et al.^35^ reported that activation of complements led to the generation of 3 anaphylatoxins: C3a, C4a, and C5a. C3a and C5a share a similar functional profile, which includes modulation of the innate and adaptive immune responses, cell homing, and tissue regeneration. In contrast, C4a has no similar function due to the absence of the C4a receptor and the inability of C4a to signal to the C3a and C5a receptors. This may also explain why C4a had no effect on osteoclasts.

**Figure 4:**
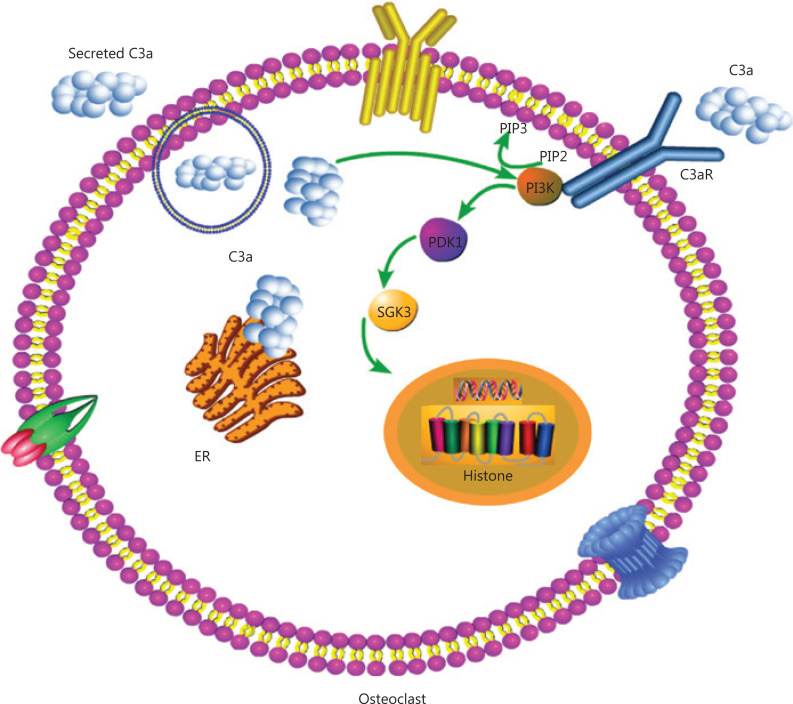
Complement C3a activates osteoclasts by regulating the PI3K/PDK1/SGK3 pathways in patients with multiple myeloma.

Based on KEGG pathway analyses, the top 4 groups were genes involved in cytokine-cytokine receptor interaction, the phospholipase D signaling pathway, the PI3K signaling pathway, and CAMs. Cytokine-cytokine receptor interaction regulates cell growth and differentiation, and is involved in immunity, inflammation, and wound healing. The phospholipase D signaling pathway participates in various cellular signal transductions. CAMs are involved in the tight and adheren junctions of antigen-presenting cells, T cells, endothelial cells, leukocytes, neurons, Schwann cells, epithelial cells, ciliary bodies, and myoblasts. The PI3K signaling pathway mainly controls cell growth, transcription and translation, cell proliferation, cell movement, and glycogen metabolism, which is most likely related to osteoclast formation, differentiation, and maturation. We selected the PI3K signaling pathway and showed that C3a promoted the formation, differentiation, and function of osteoclasts in MM patients by regulating the PI3K/PDK1/SGK3 pathway. PI3K signaling plays a central role in cellular physiology, coordinating insulin signaling during organism growth and mediating critical cellular processes, such as glucose homeostasis, protein synthesis, cell proliferation, and survival^36,37^. The PI3K signaling pathway includes PI3K-Akt signaling pathways and AKT-independent PI3K signaling pathways (including the PDK1-mTORC2-SGK axis, Rac signaling, and TEC family kinase BTK)^38^. SGKs, which consist of three isoforms, SGK1, SGK2, and SGK3, are critical mediators of AKT-independent signaling downstream of PDK1 and mTORC2 in cancer. The phosphorylation of PDK1 is activated by mTORC2, which is a multi-protein complex consisting of mTOR, Rictor, mMM Sin1, mLST8, Protor1/2, and Deptor^39^. SGK3 is a unique member within the SGK family because it contains an N-terminal PX domain, which can regulate cell processes similar to AKT kinase signaling in survival, migration, and growth signaling^40^. The amplification and overexpression of SGK3 is more common than AKT in hepatocellular carcinoma, suggesting it may have a greater functional significance in the biology of this type of cancer^41^. The activation of SGK signaling has also recently emerged as an important mechanism of resistance to PI3K and AKT inhibitors. For example, the treatment of breast cancer cells with PI3K or AKT inhibitors results in increased expression and the activation of SGK3, which depends on hVps34 for activation by PDK1 and mTORC2^42^. In our study, PDK1 was found to be an important kinase for the phosphorylation of SGK3. Thus, it is reasonable that increased PDK1 expression led to increased phosphorylation of SGK3, indicating that C3a promoted the formation, differentiation, and function of osteoclasts in NDMM patients by the PI3K/PDK1/SGK3 signaling pathways (**Figure 4**).

After treatment with a SGK inhibitor (EMD638683), up to 75% of SGK3 was inhibited at a concentration of 1 μM. In addition, the promotional effect of C3a on osteoclasts in NDMM patients was significantly inhibited. It has been reported that EMD638683 inhibits the growth and proliferation of colon carcinoma cells^43^. EMD638683 could therefore serve as a template for drugs counteracting hypertension in individuals with type 2 diabetes and metabolic syndrome^19^. In our study, treatment with EMD638683 induced a significant inhibitory effect on MM-derived osteoclasts, providing important evidence for the identification of new therapeutic targets and strategies for MBD patients. Further studies will be needed to validate its inhibitory effect on MBD in an animal model, such as mice.

## Conclusions

C3a activated osteoclasts by regulating PI3K/PDK1/SGK3 pathways in MM patients, which was reduced by treatment with a SGK inhibitor. Overall, this study identified new potential therapeutic targets and strategies for MBD patients.
