# Supplementary material for: Complement C3a activates osteoclasts by regulating the PI3K/PDK1/SGK3 pathway in patients with multiple myeloma
Source: Cancer Biol Med. 2021 Aug 15;18(3):721–33. doi: 10.20892/j.issn.2095-3941.2020.0430 (PMC8330530; doi:10.20892/j.issn.2095-3941.2020.0430)
Supplement: Supplementary file 1 [file cbm-18-721-s001.pdf]

Supplementary materials

Table S1 Primer sequences

Target genes	Sense and anti-sense sequences
OSCAR	F: 5′-GTT ACC GCT GCT GCT ACC GAA G-3′<br>R: 5′-GCG CAG GCT CAC GTT GGC-3′
RANKL	F: 5′-ATA TCG TTG GAT CAC AGC ACA TCA GAG-3′<br>R: 5′-TGT CGG TGG CAT TAA TAG TGA GAT GAG-3′
Cathepsin K	F: 5′-CCA TCC ATA ACC TTG AGG CTT CTC TTG-3′<br>R: 5′-CCA GTC ATC TTC TGA ACC ACC TCT TC-3′
TRAP	F: 5′-ATG ACC ACC TTG GCA ATG TCT CTG-3′<br>R: 5′-AGG CTG CTG GCT GAG GAA GTC-3′
SGK3	F: 5′-GGA CAG TCC AAA ACA CCA GTC A-3′<br>R: 5′-TTT CCA GAC GGT CCC AGG TT-3′
PIK3CA	F: 5′- GAG ATT GCA AGC AGT GAT AGT G-3′<br>R: 5′- TAA TTT TGG CAG TGA TTG TGG G-3′
POSTN	F: 5′- CAC CAA TGA GGC TTT TGA GAA A-3′<br>R: 5′- GAC TGC TCC TCC CAT AAT AGA C-3′
COL1A1	F: 5′- AAA GAT GGA CTC AAC GGT CTC-3′<br>R: 5′- CAT CGT GAG CCT TCT CTT GAG-3′
COL1A2	F: 5′- CTC CAT GGT GAG TTT GGT CTC-3′<br>R: 5′- CTT CCA ATA GGA CCA GTA GGA C-3′
CREB1	F: 5′- CTG ATG GAC AGC AGA TCT TAG T-3′<br>R: 5′- CTT CAT TAG ACG GAC CTC TCT C-3′
MDM2	F: 5′- CTT CTA GGA GAT TTG TTT GGC G-3′<br>R: 5′- ATG TAC CTG AGT CCG ATG ATT C-3′
IKBKG	F: 5′- GTG GAG CAC CTG AAG AGA TG-3′<br>R: 5′- CAG AGC CTG GCA TTC CTT AG-3′
NTRK2	F: 5′- CCG CAA CAA GCA CCG AGG AG -3′<br>R: 5′- CCA CAA CCA GCC AGC AGA AGC -3′
CDK2	F: 5′- CCT GGG CTG CAA ATA TTA TTC C-3′<br>R: 5′- TGG CTT GTA ATC AGG CAT AGA A-3′
TCL1A	F: 5′- GTT CGT GTA TTT GGA CGA GAA G-3′<br>R: 5′- CAG AAA CTG GAG TCT GAG GAT C-3′
AKT3	F: 5′- CAG ACA GAC TGC AGA GGC AA -3′<br>R: 5′- CCA CTT GCC TTC TCT CGA ACC -3′
PDK1	F: 5′-ACG CAC AAT ACT TCC AAG GAG ACC-3′<br>R: 5′-ATC AGC CTC GTG GTTG GTG TTG-3′
GAPDH	F: 5′-CAG GAG GCA TTG CTG ATG AT-3′<br>R: 5′-GAA GGC TGG GGC TCA TTT-3′

Table S2 Extension temperatures of target genes

Target genes	Extension temperatures
OSCAR	63.5 °C
RANKL	57.8 °C
Cathepsin K	57.8 °C
TRAP	62.3 °C
SGK3	55.4 °C
PIK3CA	57.3 °C
POSTN	57.4 °C
COL1A1	58.0 °C
COL1A2	57.8 °C
CREB1	56.0 °C
MDM2	57.1 °C
IKBKG	57.7 °C
NTRK2	64.1 °C
CDK2	57.0 °C
TCL1A	57.4 °C
AKT3	60.0 °C
PDK1	60.9 °C
